# Influence of Corneal Visualization Scheimpflug Technology Tonometry on Intraocular Pressure

**DOI:** 10.1016/j.xops.2021.100003

**Published:** 2021-01-13

**Authors:** Davide Borroni, Kunal Ajit Gadhvi, Rozaliya Hristova, Keri McLean, Carlos Rocha de Lossada, Vito Romano, Stephen Kaye

**Affiliations:** 1 Ophthalmology Department, Royal Liverpool University Hospital, Liverpool, United Kingdom; 2 Ophthalmology Department, University Hospital Aleksandrovska, Medical University of Sofia, Sofia, Bulgaria; 3 Ophthalmology Department, Malaga University Hospital, Malaga, Spain

**Keywords:** Biomechanics, CST, Glaucoma, Intraocular pressure, bIOP, biomechanically corrected intraocular pressure, CCT, central corneal thickness, CST, Corneal Visualization Scheimpflug Technology tonometry, GAT, Goldmann applanation tonometry, ICRT, Icare rebound tonometer, IOP, intraocular pressure, POAG, primary open-angle glaucoma

## Abstract

**Purpose:**

To investigate the effect of Corneal Visualization Scheimpflug Technology tonometry (CST) on intraocular pressure (IOP).

**Design:**

Cohort study.

**Participants:**

Patients with and without primary open-angle glaucoma (POAG) were included.

**Methods:**

Intraocular pressure was measured using the Icare rebound tonometer (ICRT; Icare Finland Oy) and the biomechanically corrected IOP (bIOP) using the CST. Intraocular pressure was measured at baseline with ICRT, followed by a CST measurement in one eye with the fellow eye acting as a control. Icare measurements were repeated at 10 seconds and 1, 2, 4, 8, 15, 30, and 60 minutes in both eyes. The ratio of test eye IOP to fellow eye IOP was used to control for intrasubject variation.

**Main Outcome Measures:**

Intraocular pressure change following Corneal Visualization Scheimflug Technology tonometry.

**Results:**

Forty participants (mean age, 54.09 ± 20.08 years) were included comprising 20 patients with POAG and 20 patients with no ocular abnormalities other than cataract. Mean central corneal thickness was similar in those without POAG (547.4 ± 55.05 μm) and with POAG (520.22 ± 37.59 μm; *P* = 0.14). No significant change was found in IOP measured with the ICRT in the fellow eye versus the 1-hour period in either the healthy (*P* = 0.87) or POAG (*P* = 0.92) group. Significant changes were found in IOP after CST measurement for both healthy (*P* < 0.01) and glaucomatous (*P* < 0.01) eyes. After the CST measurement, the IOP reduced continuously from a mean of 13.75 mmHg to 10.84 mmHg at 4 minutes for healthy eyes and from 13.28 mmHg to 11.11 mmHg at 8 minutes for glaucomatous eyes before approaching (83% for healthy eyes and 92% POAG eyes) the pre-CST measurement at 1 hour.

**Conclusions:**

Corneal Visualization Scheimpflug Technology tonometry causes a significant reduction in IOP in both glaucomatous and healthy eyes that lasts for at least 1 hour afterward.

Glaucoma is one of the most common causes of blindness, with more than 60 million people affected worldwide.^1^^,^^2^ Elevated intraocular pressure (IOP) remains the major modifiable risk factor for the development and progression of the disease, making it the most important therapeutic entity.^3^ Since its invention in 1954, Goldmann applanation tonometry (GAT) has been the gold standard for measuring IOP.^4^ Goldmann applanation tonometry is a contact method of tonometry based on the Imbert-Fick law of applanation and requires local anesthesia.^5^ The Icare rebound tonometer (ICRT) has gained popularity recently as a handheld device that reproducibly measures IOP, comparable with GAT, without the need for anesthesia.^6^ The ICRT calculates the IOP by measuring the rebound acceleration of a probe that is propelled perpendicularly to the patient’s cornea from a distance of 4 to 8 mm.^7^ Apart from its ease of use, ICRT has an advantage over GAT in that it does not seem to influence IOP on repeated measurement in contrast to GAT, which may induce a reduction in IOP.^8^

Despite the popularity of GAT and ICRT in clinical practice, they do not compensate for corneal biomechanics, which has led to continued interest in new developments within the field of tonometry.^9^ The Corneal Visualization Scheimpflug Technology (CST) instrument (Oculus) uses an ultra–high-speed Scheimpflug camera to visualize the corneal response to an air-puff impulse, which deforms the cornea and anterior chamber.^10^ The changes in the deformation of the cornea induced by the impulse have been used to develop a biomechanically corrected IOP (bIOP).^11^ The bIOP is thought to be less influenced by age and corneal properties such as thickness when compared with GAT or ICRT.^12^^,^^13^ However, the CST causes a significant deformation of the cornea, and it is unclear whether this leads to an alteration of the anterior chamber and IOP and how long these effects may persist. Therefore, the aim of this study was to evaluate changes in IOP after use of a CST.

## Methods

This retrospective study was conducted between April and September 2019 at The Royal Liverpool University Hospital. The study was approved by the institutional review board (identifier, A03058). The study adhered to the tenets of the Declaration of Helsinki. The requirement for informed consent was waived because of the retrospective nature of the study. Inclusion criteria were healthy patients undergoing routine ophthalmic examination and those with a diagnosis of bilateral primary open-angle glaucoma (POAG) of similar severity in both eyes. Primary open-angle glaucoma was defined as a characteristic glaucomatous progressive optic neuropathy in the absence of congenital or other ocular disease with or without raised IOP.^14^ Exclusion criteria included previous ocular surgery, secondary or angle-closure glaucoma, keratoconus or other corneal pathologic features, dense or hypermature cataract, a history of uveitis, or a combination thereof. Consecutive patients seeking treatment at the outpatient department for cataract assessment were included. The ICRT measurements were obtained 3 times in both eyes at baseline using the Icare ic100 (Icare Finland Oy) and after this, the CST was used to measure IOP once in 1 eye selected at random.

The eye that had not undergone CST served as the control. Measurement with CST was accepted as accurate when the “OK” quality index displayed on the device monitor. After CST, measurements were obtained in both eyes at 10, 60, 120, 240, 480, 900, 1800, and 3600 seconds using ICRT. Single-use probes were changed at each new examination with measurements obtained with the patient in the seated position at 90° to the corneal apex. Local anesthesia, dilating drops, or cycloplegics were not used in any of the examinations. Student *t* tests were used to compare baseline characteristics, including baseline IOP and central corneal thickness (CCT) measured using the Pachmate 2 (DGH Technology, Inc). The differences and the ratio of the test eye IOP and fellow eye IOP at baseline and at intervals after CST measurement were used to compare the 2 groups using repeated-measures analysis of variance using SPSS software version 25 (SPSS, Inc). A least squares method was used to fit curves to the changes in the ratio between the test and control eye using Maple software version 19 (Maplesoft).

## Results

Forty participants were included comprising 20 patients with an established history of POAG and 20 patients with no ocular abnormalities other than cataract. Mean age of the patients was 54.09 ± 20.08 years (range, 21–89 years). Mean CCT was similar in those without glaucoma (547.4 ± 55.05 μm) and in those with glaucoma (520.22 ± 37.59 μm; *P* = 0.14). The bIOP and IOP were measured in 21 right eyes and 19 left eyes (healthy eyes: 10 right eyes and 10 left eyes; glaucoma group: 11 right eyes and 9 left eyes). No significant difference was found in the mean bIOP and IOP for the healthy control participants (14.06 ± 2.28 mmHg and 14.62 ± 2.55mmHg, respectively; *P* = 0.14) or those with glaucoma (14.29 ± 3.22 mmHg and 14.93 ± 3.84 mmHg, respectively; *P* = 0.07).

**Figure 1:**
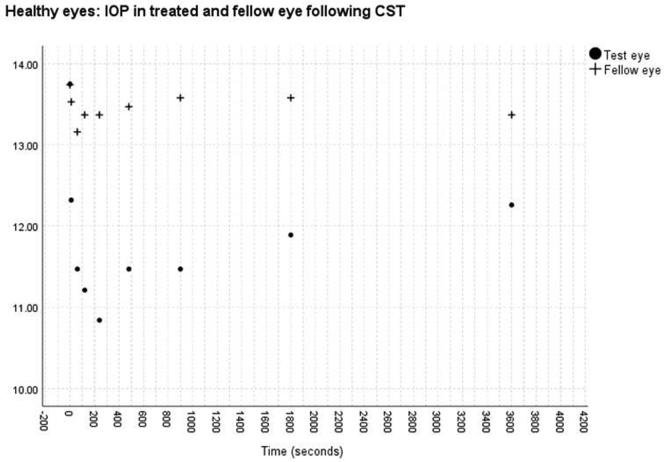
Graph showing intraocular pressure (in millimeters of mercury) in treated and fellow eye in the healthy eye group after Corneal Visualization Scheimpflug Technology tonometry measurement.

**Figure 2:**
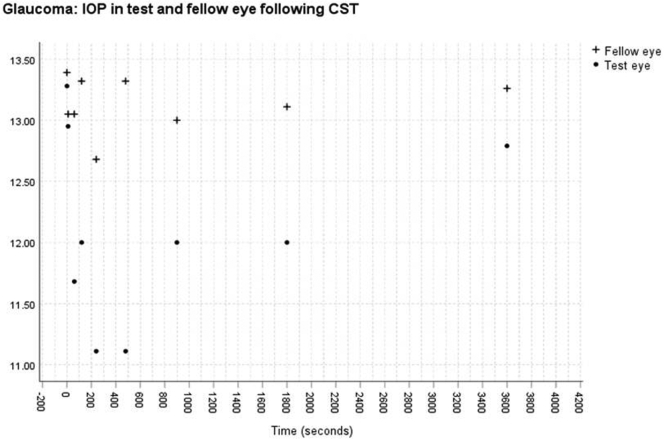
Graph showing intraocular pressure (in millimeters of mercury) in treated and fellow eyes in the glaucoma group after Corneal Visualization Scheimpflug Technology tonometry measurement.

No significant change was found in the IOP measured with the ICRT in the control eye after measurement of the bIOP in the test eye over the 1-hour period in either the healthy (*P* = 0.87) or glaucomatous (*P* = 0.92) group. Significant changes in the IOP were found after CST measurement for both healthy (*P* < 0.01) and glaucomatous (*P* < 0.01) eyes (Figs 1 and 2). After the CST measurement, the IOP reduced continuously from a mean of 13.75 mmHg to 10.84 mmHg at 4 minutes for healthy eyes and from 13.28 mmHg to 11.11 mmHg at 8 minutes for glaucomatous eyes, before approaching (83% for healthy eyes and 92% for POAG eyes) the pre-CST measurement at 1 hour (Figs 1 and 2).

**Figure 3:**
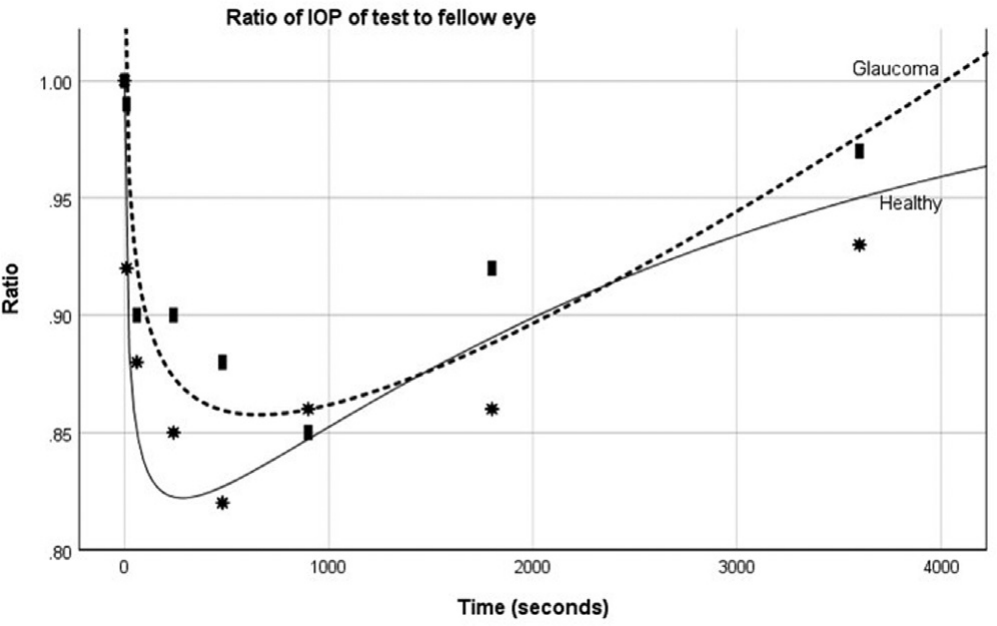
Graph showing the mean ratio at each time point of the intraocular pressure in the test eyes to that of the fellow eyes after Corneal Visualization Scheimpflug Technology tonometry measurement in healthy patients and those with primary open-angle glaucoma.

The reduction in the IOP after CST is evident as the ratio of the test eye IOP to fellow eye IOP, as shown in Figure 3. A plot of this ratio with time after the CST measurement shows that the greatest reduction in IOP occurred earlier and was greater in healthy eyes compared with glaucomatous eyes. A function of the form *a* + *bx*^*i*^ + *cx*^*j*^ + *dx*^*k*^ + *ex*^*l*^ was used to provide a best least squares curve for the ratio of the IOP for healthy and glaucomatous eyes using MapleSoft 2019. This gave

$$\text{Ratio }=\text{ 0.998 }+\;0.375\;{\left(\text{time}\right)}^{0.1}\;-\;0.701\;{\left(\text{time}\right)}^{0.25}\;+\;0.383\;{\left(\text{time}\right)}^{0.3}\;+\;4.001\text{ × }10^{-7}\;{\left(\text{time}\right)}^{1.5}$$

for eyes with glaucoma and

$$\text{Ratio }=\text{ 1.001 }+\;0.387\;{\left(\text{time}\right)}^{0.1}\;-\;0.935\;{\left(\text{time}\right)}^{0.25}\;+\;0.547\;{\left(\text{time}\right)}^{0.3}\;-\;3.25\text{ × }10^{-7}\;{\left(\text{time}\right)}^{1.5}$$

for eyes without glaucoma. Extrapolating from these equations, it is expected that the IOP after CST would return to baseline at approximately 60 minutes in glaucomatous eyes and 90 minutes in the healthy eyes.

## Discussion

Noncontact and corneal-compensated tonometry represent an expanding field of ophthalmic development,^15^ and the CST has enabled the development of a bIOP.^16^ We found that IOP measured with the ICRT and the bIOP were similar at baseline in both healthy and glaucomatous eyes and is in agreement with previous studies with average hysteresis and corneal thickness.^17^^,^^18^

However, after CST measurement, we found a significant drop in the IOP compared with the fellow eye in both healthy and glaucomatous eyes. Numerous studies have documented a reduction in IOP after GAT.^19^^,^^20^ This is likely to be multifactorial, but one explanation is that mechanical pressure on the anterior chamber caused by applanation alters the angle and increases the aqueous outflow, which subsequently reduces the IOP.^20^ Compared with GAT, the cornea and anterior chamber are deformed to a much greater extent using the CST. It is likely that this deformation alters IOP and aqueous outflow and accounts for the significant and prolonged reduction in IOP after CST.

However, it is of note that the reduction in IOP was greater and occurred earlier in the healthy versus the glaucomatous eyes. Although the patients were taking a prostaglandin analog, the magnitude of the observed effect is unlikely to be accounted for by the effect of the prostaglandin analog on corneal biomechanics.^21^ It is more probable that the difference may reflect greater resistance in outflow and or differences in scleral rigidity in eyes with glaucoma.^22^ Aqueous outflow facility is known to fluctuate and change.^23^ The relationship between IOP rise and aqueous outflow facility is not a new concept and was demonstrated in vitro in experiments involving filling of the anterior chambers of enucleated specimens.^24^^,^^25^ This relationship also was demonstrated during the same period that a rise in IOP occurs during scleral indentation followed by a reduction in IOP that is thought to be secondary to raised aqueous outflow.^26^ It is probable that a similar mechanism occurs with the CST.

For both healthy and glaucomatous eyes, the IOP had not yet returned to baseline levels by 1 hour. We did not measure the IOP after 1 hour, so it is difficult to predict when it returns to the pre-CST level. Extrapolating from the best fit curves suggests that healthy eyes take longer to reach baseline (70 minutes) than eyes with POAG (62 minutes), possibly because of the greater reduction in IOP in healthy eyes. Previous studies have demonstrated a good repeatability of bIOP using 3 consecutive CST measurements obtained at 1-minute intervals.^12^^,^^27^ This suggests that the CST induces little or no change in IOP, which is contrary to what we found in our study, or that the bIOP does not reflect the reduction in IOP. Changes in corneal curvature or the presence of corneal edema seem to influence the IOP measured on ICRT.^28^^,^^29^ We did not measure corneal curvature or thickness over the course of the study, and such alterations in these parameters may have contributed to the changes in IOP that we noted.

Because of the short interval between time points for measurement (e.g., 30 seconds, 1 minute) and changing the probe, it was not possible to repeat the ICRT at each interval. However, the Icare ic100 automatically measures the IOP 6 times, providing an average measurement only if the device deemed variation acceptable (standard deviation of measurements, <3.5 mmHg).^30^ Despite these limitations, it is clear that IOP changes significantly after use of the CST and is unlikely to return to baseline for at least 1 hour.
